# A predictive signature based on enhancer RNA associates with immune infiltration and aids treatment decision in clear cell renal cell carcinoma

**DOI:** 10.3389/fonc.2022.964838

**Published:** 2022-10-12

**Authors:** Qinyu Li, Xueyan Xiao, Bingliang Chen, Guoda Song, Kai Zeng, Beining Li, Jianping Miao, Chaofan Liu, Yang Luan, Bo Liu

**Affiliations:** ^1^ Department of Urology, Tongji Hospital, Tongji Medical College, Huazhong University of Science and Technology, Wuhan, China; ^2^ Department of Gastroenterology, Union Hospital, Tongji Medical College, Huazhong University of Science and Technology, Wuhan, China; ^3^ Department of Geriatrics, Tongji Hospital, Tongji Medical College, Huazhong University of Science and Technology, Wuhan, China; ^4^ Department of Oncology, Tongji Hospital, Tongji Medical College, Huazhong University of Science and Technology, Wuhan, China

**Keywords:** clear cell renal cell carcinoma, drug sensitivity, enhancer RNA, immunotherapy, treatment decision

## Abstract

Clear cell renal cell carcinoma (ccRCC) is a prevalent urinary malignancy. Despite the recent development of better diagnostic tools and therapy, the five-year survival rate for individuals with advanced and metastatic ccRCC remains dismal. Unfortunately, ccRCC is less susceptible to radiation and chemotherapy. Consequently, targeted therapy and immunotherapy play a crucial role in the treatment of ccRCC. Enhancer RNAs (eRNAs) are noncoding RNAs transcribed by enhancers. Extensive research has shown that eRNAs are implicated in a variety of cancer signaling pathways. However, the biological functions of eRNAs have not been systematically investigated in ccRCC. In this study, we conducted a comprehensive investigation of the role of eRNAs in the onset and management of ccRCC. Patient prognosis-influencing eRNAs and target genes were chosen to construct a predictive signature. On the basis of the median riskscore, ccRCC patients were split into high- and low-risk subgroups. The prediction efficiency was assessed in several cohorts, and multi-omics analysis was carried out to investigate the differences and underlying mechanisms between the high- and low-risk groups. In addition, we investigated its potential to facilitate clinical treatment choices. The riskscore might be used to forecast a patient’s response to immunotherapy and targeted therapy, giving a revolutionary method for selecting treatment regimens with pinpoint accuracy.

## Introduction

Renal cancer is one of the most common malignant tumors, and the estimated numbers of new deaths and new cases of renal cancer in the United States in 2021 were 13,780 and 76,080, respectively (1). Renal cell carcinoma (RCC) represents 90% of renal malignancies, with clear cell renal cell carcinoma (ccRCC) being the most prevalent subtype (2). Currently, the most effective therapy for ccRCC is surgery. However, following surgery, relapses and metastases are prevalent (3). Over 30% of ccRCC patients are reportedly affected by metastatic disease (4). Despite advancements in cancer detection and treatment, the 5-year survival rate for individuals with metastatic ccRCC remains around 20% (5). Since ccRCC does not respond well to radiation and chemotherapy, targeted therapy and immunotherapy become particularly important in the treatment of ccRCC (6, 7). Evidently, a customized strategy is crucial. On the basis of both the features of carcinomas and the situations of patients, the treatment regimen should be developed comprehensively.

Immunotherapies, particularly immune checkpoint blockade (ICB), have changed cancer therapy in recent years (8). Certain individuals with ccRCC exhibit astonishing clinical improvements when immune suppression is eliminated by ICB, and in the treatment of advanced ccRCC, checkpoint blockade in combination with other anticancer drugs is presently the first-line therapy (9). However, a substantial proportion of patients do not qualify for checkpoint blockade. This necessitates the use of reliable biomarkers to determine the optimal therapy strategy.

Enhancers are short clusters of regulatory DNA elements with transcription factor recognition sequences. They are either close to or far from the promoters of the target genes and control their expression (10, 11). Enhancers have been shown to play crucial roles in cancer formation and tumor response (12–14). Beyond their typical role of recruiting transcription factors to gene promoters, enhancer elements are also transcribed into noncoding RNAs known as enhancer RNAs (eRNAs) (15). eRNAs appear to be engaged in several cancer signaling pathways by modulating the expression of their target genes, including clinically actionable genes and immune checkpoints (16). By interacting with transcription factors, eRNAs may actually boost therapeutic anticancer therapies (16–18). eRNAs may serve as diagnostic indicators and tissue-specific treatment targets due to their tissue-specific expression across various cancer types. The risk model has been constructed in glioma (19), prostate cancer (20), and hepatocellular carcinoma (21). However, to the best of our knowledge, how eRNAs affect immune function and influence survival outcomes in ccRCC patients remains to be investigated.

In this study, we conducted an integrated analysis to investigate the functions of eRNAs in the development and management of ccRCC. Patient prognosis-influencing eRNAs and target genes were chosen to generate a predictive signature. Multi-omics analysis was performed to evaluate the differences and underlying processes between the high- and low-risk groups. Prediction accuracy was assessed in several cohorts. In addition, we explored the correlations of patient responsiveness to immunotherapy and targeted therapies with riskscore to evaluate its potentiality to facilitate treatment choices in clinical practice.

## Materials and methods

### Data collection and preprocessing

From the TCGA database, transcriptional data, somatic mutation data, and clinical features were obtained (**Table S1**). Then, we obtained the following datasets from the GEO database: GSE53757 (22) (N=144), GSE46699 (23) (N=130), GSE36895 (24) (N=76), and GSE22541 (25) (N=68). By using the “ComBat” algorithm of the “sva” package (26), we eliminated the batch effect and pooled the four datasets as a validation cohort. As further confirmation, we also retrieved RNA-seq data from the ICGC-RECA-EU database. The E-MTAB-1980 cohort (27) from the ArrayExpress database was also used to verify the predictive value.

### Identification of prognostic eRNAs in ccRCC

PreSTIGE (Predicting Specific Tissue Interactions of Genes and Enhancers) was used to investigate lncRNAs produced from active tissue-specific enhancers and their putative target genes. We obtained lncRNA−target gene association data from a previous study for further analysis (28). To recognize the prognostic eRNAs, univariate Cox regression was used to determine candidate eRNAs and their respective target genes. After analysing the connection between eRNAs and target genes (Cor > 0.30, P-value< 0.01), we applied LASSO regression analysis for further screening. Finally, a multivariate Cox regression analysis (P-value< 0.05) was utilized to further identify eRNAs playing important roles in the prognosis of patients.

### Establishment and evaluation of the prognostic signature

The prognostic model was constructed according to the results of multivariate Cox regression analysis. The riskscore for each patient was calculated by applying the following formula:


$$\text{Riskscore =}\sum_{i=1}^{n}\beta i*expi$$

*β represents the regression coefficient, exp represents the expression value of each eRNA

On the basis of the median riskscore, ccRCC patients in the TCGA and E-MTAB-1980 cohorts were split into high- and low-risk subgroups. Using Kaplan-Meier curves, survival disparities between the high- and low-risk groups were displayed. Moreover, the “timeROC” package (29) was applied to assess the prognostic efficacy of the signature. To uncover the differences between the high- and low-risk groups, differential expression analysis was performed with the parameters of |logFC| > 1 and P< 0.05. Furthermore, GO and KEGG enrichment analyses were carried out by the R package “clusterProfiler” (30) to unearth the potential mechanism (FDR< 0.05 and q value< 0.05).

### Correlation between riskscore and clinical parameters

To elucidate the influence of the riskscore on cancer development, the difference in riskscore across patients stratified by clinical parameters was calculated. We examined the percentage of patients in various stages between the high- and low-risk groups and investigated the predictive validity of the riskscore in early-stage and late ccRCC patients, given that pathologic stage is crucial for therapy selection. In addition, univariate and multivariate Cox regression analyses were conducted to assess the independence of the prognostic model from other clinicopathological characteristics.

### Tumor mutation burden analysis

Tumor mutation burden (TMB) has been shown to be a predictive biomarker for determining whether cancer patients would react favorably to immune checkpoint inhibitors (31). Therefore, we investigated the relationship between TMB and riskscore in this study. Furthermore, patients were separated into four categories based on the median riskscore and TMB. The Kaplan-Meier method was used to evaluate differences in the survival distributions between groups.

### Correlation between immune infiltration and riskscore

Using the “GSVA” R package, we compared the infiltration levels of 28 immune cells and immunological functioning across high- and low-risk groups (32). The Immune score and Stromal score of each sample were calculated by the “ESTIMATE” package (33). The correlations between immune infiltration and riskscore were then calculated after downloading the infiltration estimate data predicted by CIBERSORT, TIMER, xCell, quanTIseq, MCP-counter, and EPIC from TIMER 2.0 (34). In addition, the immunological subtypes of patients in the high- and low-risk groups were compared. Finally, a comparison was made between the riskscore and the human leukocyte antigen (HLA) gene family.

### Benefits of the riskscore to aid treatment decision

Immunotherapy for cancer has altered the standard of treatment for certain patients with advanced disease (35). ICB has received attention in ccRCC patients as a promising anticancer treatment. Here, we first evaluated the differential expression of several immune checkpoint genes across high- and low-risk groups. Furthermore, we evaluated the correlations between CTLA4 and PD-1 expression and riskscore in different stages. Additionally, we retrieved the immunophenoscore (IPS) from The Cancer Immunome Database (TCIA) for ccRCC patients. The patient’s IPS was determined objectively by taking into account the four categories of immunogenicity-determining genes: effector cells, immunosuppressor cells, MHC molecules, and immune modulators (36). Immunogenicity is positively linked with higher IPS scores (37). In addition, we downloaded TCGA patient analysis findings from the Tumor Immune Dysfunction and Exclusion (TIDE). Three cell types believed to inhibit T-cell infiltration in tumors were studied across high- and low-risk groups: cancer-associated fibroblasts (CAFs), myeloid-derived suppressor cells (MDSCs), and the M2 subtype of tumor-associated macrophages (TAMs). Due to the absence of publicly available data on ccRCC cohorts adopting immunotherapy, we employed an immunotherapeutic cohort (IMvigor210 cohort) as a validation cohort (38). Comparisons of riskscore were made between patients with various clinical states after therapy.

Given that VEGFR-targeted therapy remains the first-line therapeutic choice for advanced ccRCC, we compared the susceptibility to sorafenib, sunitinib, pazopanib, and axitinib across high- and low-risk groups. The half maximal inhibitory concentrations (IC50) of these drugs were compared by applying the pRRophetic (39) package. In addition to the TCGA cohort, the findings were verified in the GEO and ICGC cohorts as well.

### Statistical analysis

All of the analyses were conducted using RStudio 4.0.4. The Spearman method was used to calculate correlations. Two groups were compared using Student’s t test (mean with SD) and the Wilcoxon test. Multiple groups were analyzed using the Kruskal–Wallis test and one-way ANOVA. Statistical significance was defined as P-value< 0.05.

## Results

### Identification of prognosis-associated eRNAs and target genes in ccRCC

**Figure 1:**
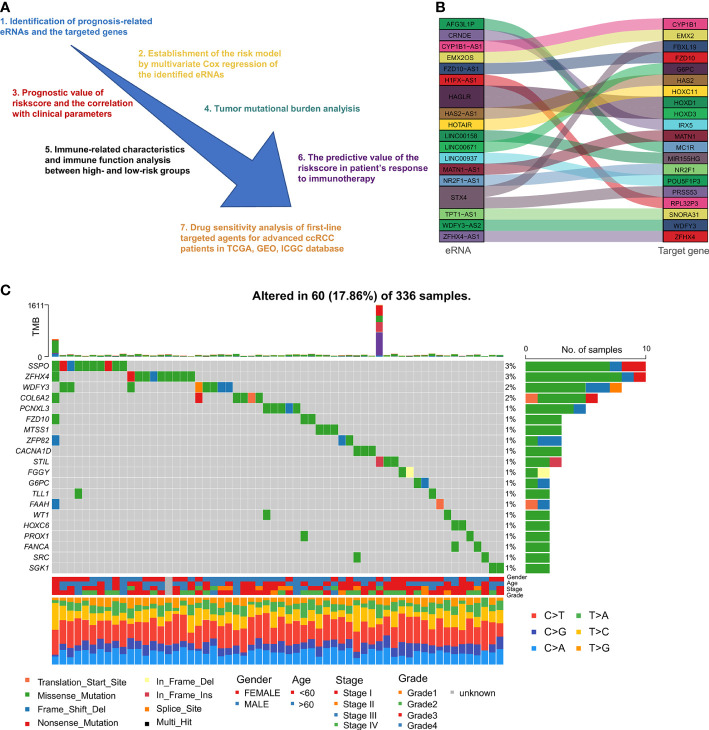
Flow chart and identification of prognostic eRNAs: **(A)** Diagram showing the methodology of this study. **(B)** Top 20 correlated eRNAs and their target genes relevant to survival. **(C)** The top 20 mutated eRNAs or target genes associated with patient prognosis.

**Figure 1A** illustrates the research design for this study. We obtained 10066 regulatory relationships of eRNA and its target gene from previous research (28). After univariate Cox regression analysis and correlation analysis, 85 paired eRNAs and their target genes were identified (**Table S2**), including 69 eRNAs and 80 target genes. The corresponding relationship between the top 20 eRNAs associated with survival and their target genes is shown in **Figure 1B**. Moreover, we displayed the top 20 eRNAs or target genes with the highest mutation frequency (**Figure 1C**).

### Construction of the predictive signature and the prognostic value

**Figure 2:**
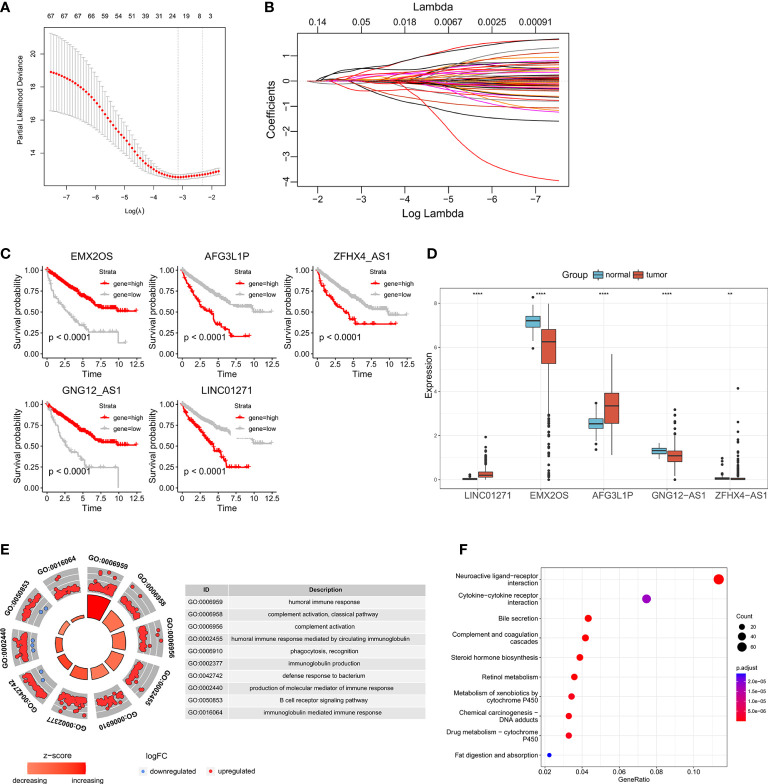
Identification of prognosis-associated eRNAs: **(A, B)** LASSO regression analysis of the eRNAs correlated with OS in univariate Cox regression analysis. Survival analysis **(C)** and differential expression analysis **(D)** of the five eRNAs constructing the predictive signature. **(E, F)** GO and KEGG analyses between the high- and low-risk groups. (**p < 0.0, ****p < 0.0001).

The 69 eRNAs were filtered by using LASSO regression analysis (**Figures 2A, B** **)**. The number of potential eRNAs was reduced to eight. After multivariate Cox regression analysis, five eRNAs (EMX2OS, GNG12-AS1, ZFHX4-AS1, AFG3L1P, LINC01271) remained to construct the predictive signature. The correlations between the five eRNAs are shown in **Figure S1**. The survival analysis and differential analysis between tumor and normal tissues performed well in terms of the prognostic genes (**Figures 2C, D** **)**. Thus, we calculated the riskscore of each patient as follows: riskscore = (-0.1284 × EMX2OS expression) + (0.3209 × AFG3L1P expression) + (0.3546 × ZFHX4-AS1 expression) + (-0.8311 × GNG12-AS1 expression) + (0.9646 × LINC01271 expression) (**Table 1**).

**Table 1:** Multivariate Cox regression analysis to identify prognosis-related eRNAs.

eRNA	coef	exp (coef)	se (coef)	z	Pr(>|z|)
EMX2OS	-0.1284	0.8795	0.0560	-2.2939	0.0218
AFG3L1P	0.3209	1.3784	0.1203	2.6677	0.0076
ZFHX4-AS1	0.3546	1.4256	0.1550	2.2884	0.0221
GNG12-AS1	-0.8311	0.4356	0.2264	-3.6711	0.0002
LINC01271	0.9646	2.6237	0.3853	2.5036	0.0123

Coef, coefficient.

**Figure 3:**
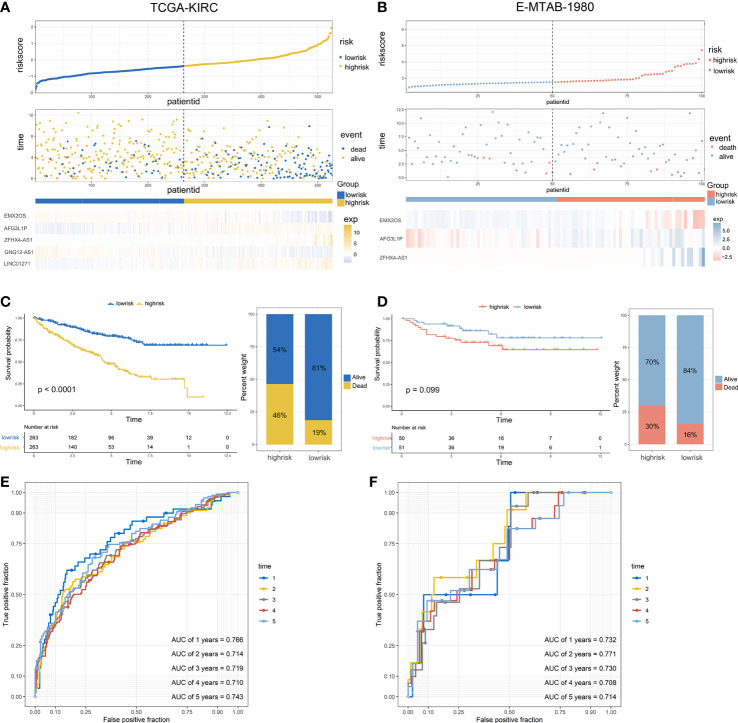
Validation of the predictive signature and performance analysis: **(A, B)** The riskscore distribution, survival status, and the expression of five eRNAs of patients in the TCGA cohort and E-MTAB-1980 cohort. **(C, D)** Kaplan-Meier survival curves of OS and the proportion of the survival rate between the high- and low-risk groups of the two datasets. **(E, F)** Time-dependent receiver operating characteristic analysis of the two datasets.

After generating the riskscore, we conducted assessment and validation analyses. First, ccRCC patients from the TCGA-KIRC and E-MTAB-1980 cohorts were separated into high- and low-risk groups (**Table S3**), and the survival status of each patient in the cohort assessed by riskscore is shown in **Figure 3A, B**. The high-risk group had a greater mortality rate than the low-risk group, and Kaplan-Meier analysis indicated substantial differences in overall survival between the high-risk and low-risk groups (**Figure 3C, D**). Additionally, the predicted signature’s accuracy was also assessed using a time-dependent receiver operating characteristic curve analysis. The AUCs for 1-year, 2-year, 3-year, 4-year, and 5-year survival in the TCGA-KIRC dataset were 0.766, 0.714, 0.719, 0.710, and 0.743, respectively (**Figure 3E**). The E-MTAB-1980 cohort had AUCs of 0.732, 0.771, 0.730, 0.708, and 0.714, respectively, for survival rates at one, two, three, four, and five years (**Figure 3F**).

To investigate the biological differences between the high- and low-risk groups. GO and KEGG enrichment analyses were carried out to identify the biological functions of differentially expressed genes (**Figures 2E, F** **)**. We discovered that the majority of the biological processes were immune system functions, such as humoral immune response, complement activation, and phagocytosis. Analysis of KEGG pathways indicated that differentially expressed genes were mostly engaged in neuroactive ligand-receptor interaction and cytokine-cytokine receptor interaction.

### Correlation between the predictive signature and clinical parameters

**Figure 4:**
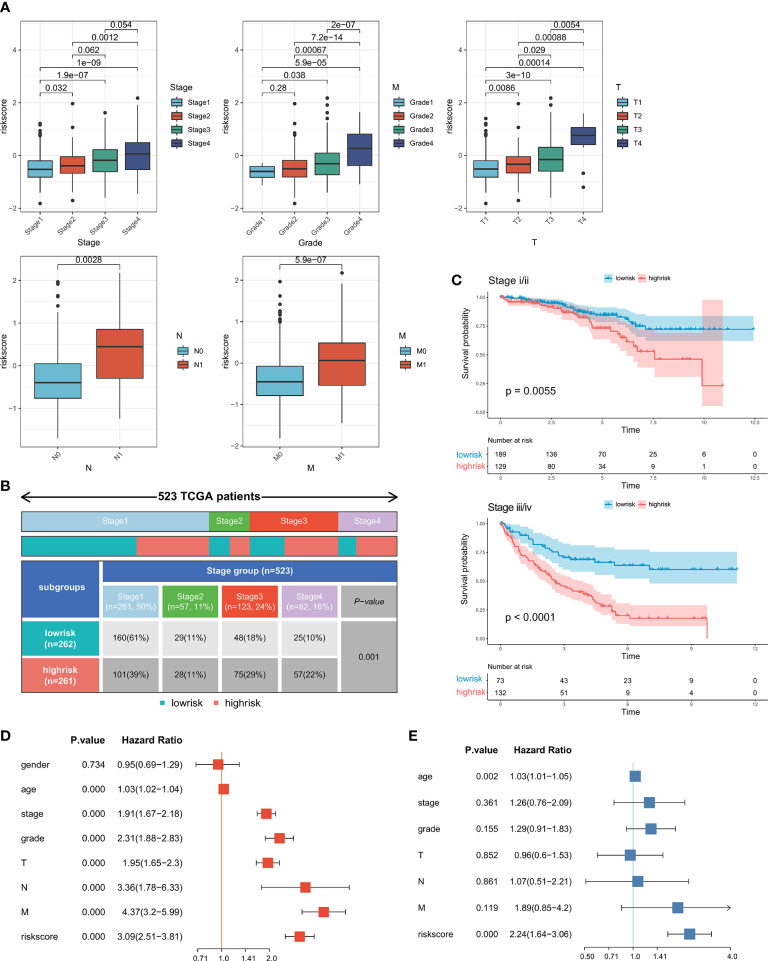
Association between the predictive signature and clinical parameters: **(A)** Correlations between riskscore and progression of ccRCC. **(B)** The proportion of patients in different stages between the high- and low-risk groups. **(C)** Kaplan-Meier survival curves of OS in early and advanced patients. **(D)** Univariate and **(E)** multivariate Cox regression analyses of correlations between the riskscore and clinical parameters.

To investigate the link between the predictive signature and clinical parameters, we evaluated the riskscore verified by clinical parameters, and the results indicated that the riskscore was markedly increased in patients with advanced pathologic stage, histologic grade and TNM stage (**Figure 4A**). Due to the importance of the pathologic stage in determining the optimal therapy, we examined the percentage of patients at various stages between the high- and low-risk groups. We discovered that the percentage of advanced patients in the high-risk category was dramatically increased (**Figure 4B**). (**Figure 4B**). We also evaluated the predictive validity of the riskscore in patients with early and advanced ccRCC (**Figure 4C**). We observed that the predictive signature could differentiate patient prognosis in both early and advanced stages well.

To determine whether the predictive signature was an independent prognostic indicator in ccRCC, univariate and multivariate analyses were performed. The HR of the riskscore was 3.09 (95% CI: 2.51-3.81) and 2.24 (95% CI: 1.64-3.06) (**Figures 4D, E** **)**, respectively, indicating that the riskscore was an independent prognostic factor in ccRCC. Interestingly, in the multivariate analyses, pathologic stage, histologic grade and TNM stage showed no significant differences when combined with riskscore, which also demonstrated a tight correlation between the predictive signature and clinical parameters.

### Relationship between riskscore and TMB

**Figure 5:**
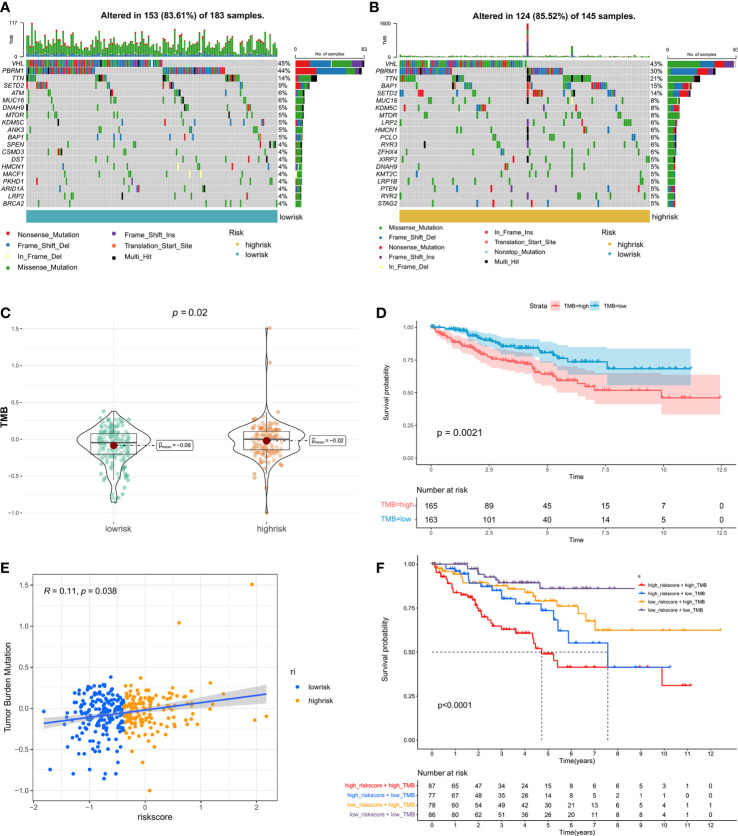
The correlation between TMB and riskscore: Genomic mutation status in the low-risk **(A)** and high-risk **(B)** groups is shown *via* a waterfall graphic. **(C)** The difference in TMB between groups with high- and low-risk. **(D)** The PFS survival curve between the high- and low-TMB groups. **(E)** Correlation analysis of riskscore and TMB. **(F)** Graphs showing Kaplan-Meier survival curves for four subgroups stratified by risk scores and TMB.

TMB is emerging as a promising biomarker for predicting patients’ immune checkpoint inhibitor responses (40). Here, we assessed the relationship between riskscore and TMB. Patients in the high-risk group had a higher TMB than those in the low-risk group (**Figure 5C**), and patients with a higher TMB had a shorter PFS (**Figure 5D**). In addition, the correlation analysis revealed that the riskscore was positively related to TMB (**Figure 5E**). Then, patients were separated into four groups based on the riskscore and TMB. Patients in riskscore^high^TMB^high^ had the shortest median PFS, whereas those in riskscore^low^TMB^low^ had the best prognosis (**Figure 5F**). Finally, a graphic representation of the mutation status of genes that had high mutation rates in the high- and low-risk categories was visualized (**Figures 5A, B** **)**.

### Riskscore was associated with ccRCC immune infiltration

**Figure 6:**
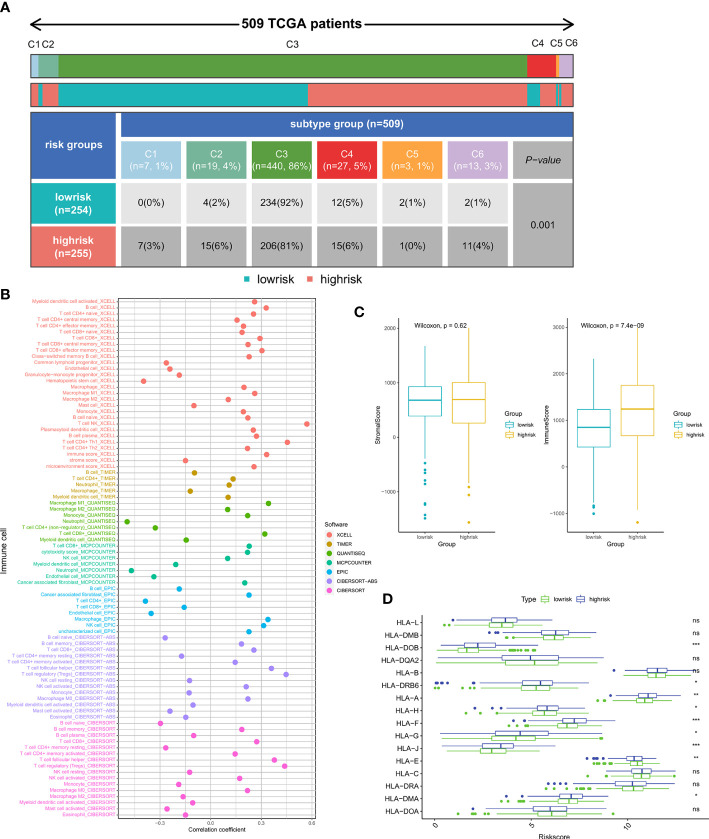
Relationship between immune infiltration and riskscore: **(A)** The proportion of patients with different immune subtypes between the high- and low-risk groups. **(B)** The correlation of the riskscore and immune cell infiltration levels. **(C)** Comparison of Immune score and Stromal score between the high- and low-risk subgroups. **(D)** The expression of the HLA gene family between the high- and low-risk groups. (*p < 0.05, **p < 0.01, ***p < 0.001). ns, no significance.

We then explored the possible association between the riskscore and the tumor immunity of patients. In the high-risk and low-risk groups, there was a significant difference between the levels of most immune cells infiltrated (**Figure S2**) and the functions of these cells (**Figure S3**). Furthermore, we compared the levels of immune cell infiltration with the riskscore, and the findings indicated that the riskscore was closely associated with the majority of the immune cells (**Figure 6B**). Immune subtypes are characteristics of the tumor microenvironment (TME) that cut beyond conventional cancer classifications to generate groups and imply that some therapeutic methods may be independent of histologic type. We retrieved immune subtypes of TCGA from a previous work (41) and compared the prevalence of various immunological subtypes in high- and low-risk groups. The findings demonstrated striking changes in immunological properties between the high- and low-risk groups (**Figure 6A**). As shown in **Figure 6C**, the Immune Score of the high-risk patients was significantly higher than that of the low-risk patients. In addition, the expression levels of the majority of HLA-related genes were elevated in the high-risk groups (**Figure 6D**).

### Riskscore predicts therapeutic benefits

**Figure 7:**
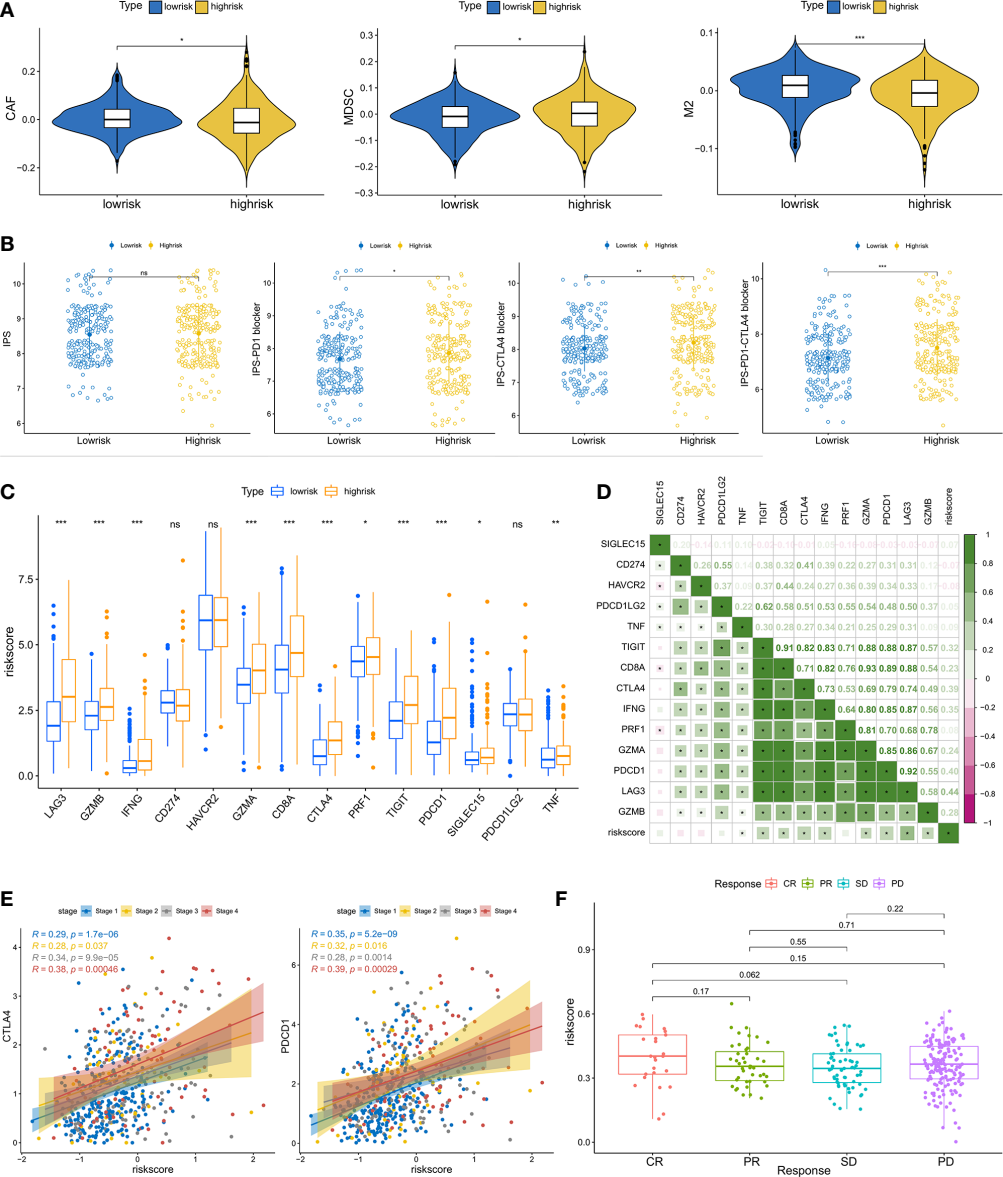
Predictive value of riskscore for immunotherapy: **(A)** The infiltration levels of CAFs, MDSCs, and M2-TAMs between the high- and low-risk groups. **(B)** The correlation between IPS and riskscore. **(C)** The expression of immune checkpoint genes in subgroups with high- and low-risk. **(D)** The association between riskscore and immune checkpoint gene expression. **(E)** The correlation between CTLA4, PD-1 expression and the riskscore in different pathologic stages. **(F)** The difference in riskscore among individuals in the IMvigor210 cohort performing different responses to immunotherapy. (*p < 0.05, **p < 0.01, ***p < 0.001). ns, no significance.

Anticancer immunotherapies using inhibitors of immune checkpoints have been developed as novel treatment regimens (42). The tumor microenvironment (TME) has been shown to be closely connected to tumor growth and metastasis (43) and may dampen the treatment response, hence influencing the clinical outcome (44). To further investigate the relationships between immunotherapy response and riskscore, we analyzed the association between riskscore and immune checkpoint inhibitor genes. The expression of ICI genes, including CTLA4 and PD-1, was elevated in the high-risk groups relative to the low-risk groups (**Figure 7C**). The correlation analysis also suggested that the riskscore was positively correlated with immune checkpoint gene expression (**Figure 7D**). Considering that immunotherapies are typically prescribed to patients with advanced ccRCC, we also estimated the correlation between riskscore and immune checkpoint gene expression across different stages. It was observed that the correlation coefficient was increased in advanced status, especially in stage iv (**Figure 7E**). IPS analysis was used to assess the immunogenicity of the two prognostic groupings. Patients in the high-risk group had higher IPS, IPS-CTLA4, IPS-PD1, and IPS-PD1-CTLA4 scores, suggesting that immunotherapy may have a greater response in this group (**Figure 7B**). CAFs, MDSCs and M2-TAMs have been shown to inhibit T-cell infiltration into cancers, and we calculated their prevalence in prostate cancer. High-risk individuals had considerably fewer CAFs and M2-TAMs (**Figure 7A**). However, the high-risk group had a larger percentage of MDSCs. Finally, we examined the riskscore performance in the IMvigor210 cohort. We compared the riskscore of patients with different immunotherapy responses. Patients with the greatest riskscore were those who had a complete response (**Figure 7F**). According to these findings, patients in the high-risk category may benefit from immunotherapy.

**Figure 8:**
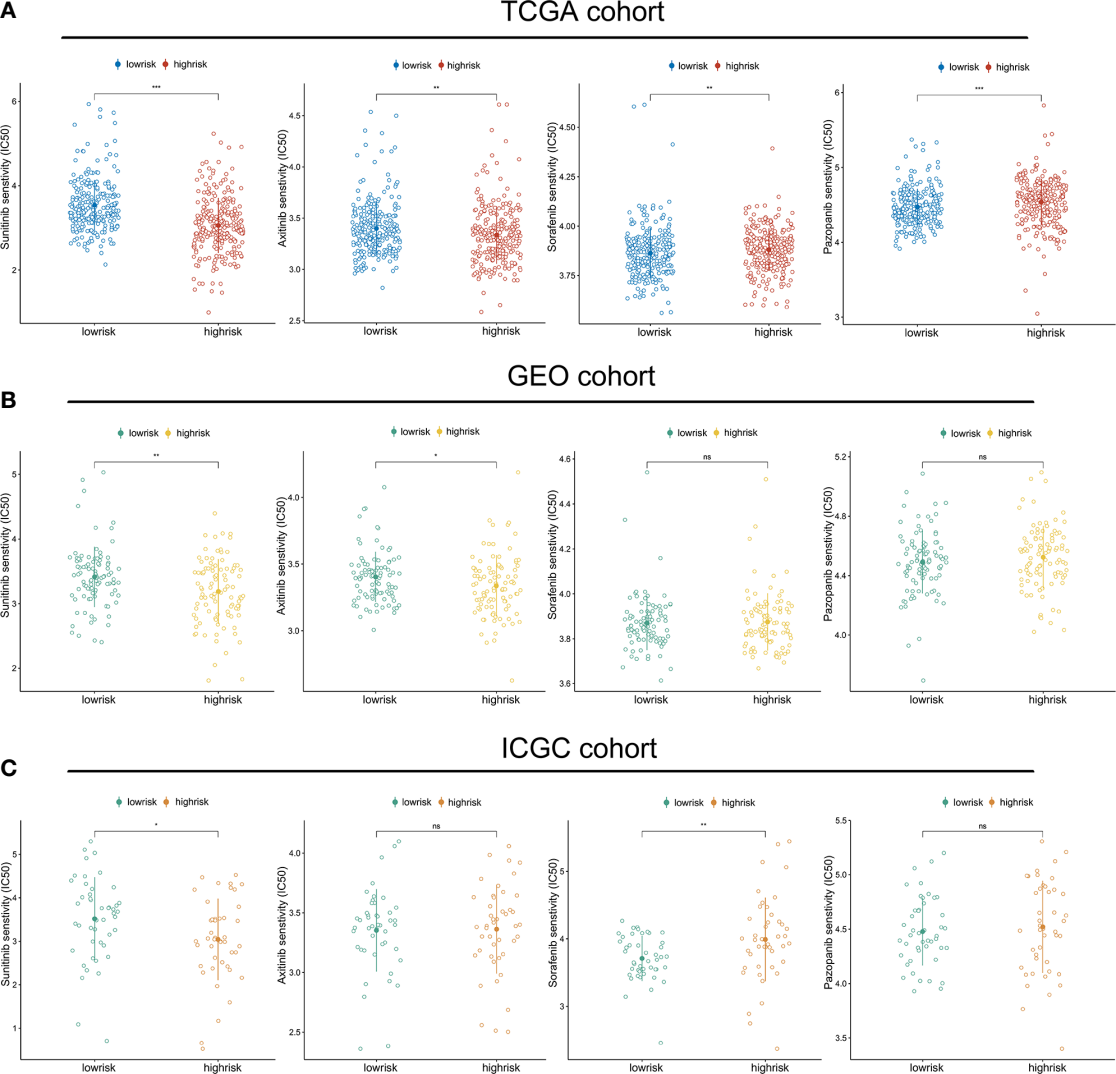
Riskscore predicts therapeutic benefits of targeted therapies: The estimated IC50 values of sunitinib, axitinib, sorafenib and pazopanib in the TGCA cohort **(A)**, GEO cohort **(B)**, and ICGC cohort **(C)**. (*p < 0.05, **p < 0.01, ***p < 0.001). ns, no significance.

Given that VEGFR-targeted therapy remains the first-line therapeutic choice for advanced ccRCC, we compared the sensitivity of sorafenib, sunitinib, pazopanib, and axitinib across high- and low-risk groups. In the TCGA cohort, we discovered that the high-risk group was more likely to respond to sunitinib and axitinib, while the low-risk group responded better to sorafenib and pazopanib (**Figure 8A**). The validation cohort was subsequently used to verify these findings. The GEO cohort confirmed the sensitivities to sunitinib and axitinib for patients with a high riskscore (**Figure 8B**), while the ICGC cohort validated the sensitivities to sunitinib and resistance to sorafenib (**Figure 8C**) (**Table S4**).

## Discussion

The most prevalent type of kidney cancer is ccRCC. Due to the absence of accurate diagnostic biomarkers and the constraints of efficient screening detection, about 30% of RCC patients first present with metastatic disease (45). Recurrence occurs in around 30% of individuals after full excision of the initial tumor (46). Traditional chemotherapy and radiation therapy are generally unsuccessful against all subtypes of RCC (47). Lack of susceptibility to chemotherapy and radiation therapy has motivated study into other therapeutic methods. In recent years, the introduction of targeted therapies, such as multi-targeted tyrosine kinase inhibitors and mTOR inhibitors, has been a significant advancement in ccRCC treatment (7).

Recent studies indicates that the immune microenvironment generated by tumor immune cells may control the development of cancer (48–50). Immune checkpoint inhibitors have emerged as viable therapy choices for advanced ccRCC in recent years (51–53). Immunotherapies such as nivolumab have already been included in metastatic ccRCC therapy regimens (2). However, in the setting of high tumor heterogeneity in terms of cell biological characteristics and genetics, metastatic or advanced RCC patients still have a poor prognosis due to the absence of effective therapeutic approaches that may produce long-lasting responses (54, 55). Therefore, identifying and validating biomarkers are essential for enhancing first-line selection and treatment sequences (56).

Recent breakthroughs in genome-wide investigations have shown that the dysregulation of distal gene regulatory elements, such as enhancers, occurs in a number of pathophysiological diseases (57). They generate eRNAs, which directly contribute to tumorigenesis (58, 59). Through their ability to resolve intratumor heterogeneity with specificity of cell-type enhancers, eRNAs provide additional explanations for cancer phenotypes beyond those provided by mRNA expression (60). In addition to regulating gene expression in cancer, eRNAs also maintain genome stability, which is associated with chromosome rearrangements and genome instability (61). Moreover, it has been shown that eRNAs are expressed across human cancer tissues, indicating that they could be useful as biomarkers and therapeutic targets (62). Cancer resistance is a well-defined phenomenon that arises when cancer cells develop tolerance to a certain therapeutic dosage. Several molecular pathways, including genetic or epigenetic changes and enhanced drug efflux, contribute to therapeutic resistance (57). Deregulation of enhancer transcription has been linked to various malignancies and their treatments during the last decade (63, 64). However, the prognostic significance of eRNAs in ccRCC is yet unknown.

In this study, active tissue-specific enhancers and their predicted potential target genes were obtained from previous research. The prognostic eRNA and its target gene were filtered using univariate Cox regression analysis and correlation analysis. Next, LASSO regression analysis and multivariate Cox regression analysis were used to build the predictive signature. Finally, five eRNAs (EMX2OS, GNG12-AS1, ZFHX4-AS1, AFG3L1P, and LINC01271) remained to construct the predictive signature. The prognostic value of riskscore was demonstrated in both the TCGA and E-MTAB-1980 cohorts. In addition, the clinical correlation study revealed a positive association between the riskscore and T stage, N stage, M stage, histologic grade, and pathologic stage. In the high-risk group, the percentage of advanced patients was much higher. GO and KEGG analyses were carried out to identify the likely underlying mechanism. We observed that the biological processes enriched by differentially expressed genes were highly connected with the immune response, suggesting that there may be substantial variations between the high- and low-risk groups in terms of immunological features.

Multiple recent studies have shown that TMB correlates with immunotherapy response because it reflects the total neoantigen load (65, 66). Low TMB is a good prognostic marker but predicts the adverse predictive effectiveness of ICI therapy (65). Herein, we discovered a substantial positive association between the riskscore and TMB. Patients in the high-risk group had a higher TMB, indicating a more favorable treatment response. In addition, the expression levels of the majority of HLA-related and immune checkpoint genes were considerably elevated in the high-risk groups. Interestingly, we found that the correlation coefficient was higher in individuals with advanced disease, particularly those diagnosed with stage IV. Therefore, high-risk individuals with advanced disease may have a higher expression of CTLA4 and PD-1. Higher scores on the IPS are related to increased immunogenicity (36). In the high-risk group, the IPS, IPS-CTLA4, IPS-PD1, and IPS-PD1-CTLA4 values were considerably higher. Thus, patients in the high-risk group may exhibit a stronger immunotherapy response. Due to the rarity of open-access data on ccRCC cohorts receiving immunotherapy, we utilized IMvigor210 cohort patients for preliminary validation. Consistent with the aforementioned hypotheses, we discovered that patients who had a complete response had the highest risk score.

Given the importance of targeted therapy for patients with advanced or metastatic ccRCC, we compared the IC50 values of the first-line agents between the high- and low-risk groups. In the TCGA cohort, we found that the high-risk group was more likely to be responsive to sunitinib and axitinib, while the low-risk group was more sensitive to sorafenib and pazopanib. These findings were confirmed in the GEO and ICGC cohorts. Obviously, the combination of targeted therapies and personalized immunotherapy is more suited for patients with advanced ccRCC, since it is unreasonable to expect that a single treatment can significantly improve their prognosis. In fact, according to the recommendations of the European Association of Urology, the combination of nivolumab and ipilimumab or pembrolizumab and axitinib are the new first-line therapy for patients at intermediate and poor risk (67). It has been observed that pembrolizumab plus axitinib therapy resulted in substantially higher overall survival and progression-free survival than sunitinib treatment among patients with advanced renal-cell carcinoma who had not been treated before (68). Another trial also recognized that progression-free survival was considerably longer with avelumab plus axitinib compared to sunitinib in patients with advanced renal cell carcinoma (69). Actually, targeted metastatic RCC treatments have been demonstrated to have immunomodulatory effects, such as raising tumor cell antigenicity and encouraging T-cell infiltration (70). These results sparked an interest in exploring the possibility of combining targeted antiangiogenic medicines with immunotherapies to maximize any potential synergies (71). In this study, it was shown that high-risk individuals respond better to immunotherapy and are more sensitive to sunitinib and axitinib. On the basis of these results, we hypothesize that the riskscore might be used to aid in the selection of treatment regimens in practical practice. The combination of sunitinib, axitinib, and immunotherapy may be beneficial for high-risk individuals, while sorafenib and pazopanib are more effective for low-risk patients.

Although we constructed a predictive signature to aid treatment decision in ccRCC, several limitations to this study need to be acknowledged. First, eRNA is a subtype of noncoding RNA produced by enhancers, and it is not or is rarely detected in many RNA-seq datasets. Therefore, validation in the GEO cohort and E-MTAB-1980 cohort could not include all the eRNAs in the signature, which may reduce the efficiency of the signature. In addition, due to the paucity of open-access immunotherapy cohort data for ccRCC, a preliminary validation was conducted in the IMvigor210 cohort of bladder cancer. The potential of this signature to predict the immunotherapy response of patients in ccRCC groups receiving treatment requires additional confirmation.

## Conclusions

In summary, we conducted an exhaustive investigation of the involvement of eRNAs in the development and treatment of ccRCC. The prognosis-influencing eRNAs and target genes were chosen to generate a predictive signature. Multi-omics analysis was performed to examine the differences and underlying mechanisms between the high- and low-risk groups, and the predictive accuracy was assessed in multiple cohorts. In addition, relationships between patient response to immunotherapy, targeted therapies, and riskscore were assessed. Our findings indicated that the combination of sunitinib, axitinib, and immunotherapy may be beneficial for high-risk individuals, while sorafenib and pazopanib are more effective for low-risk patients.

## Data availability statement

The original contributions presented in the study are included in the article/**Supplementary Material**, further inquiries can be directed to the corresponding authors.

## Author contributions

QL and XX designed the study and wrote the manuscript. CL, YL and BL provided administrative support. BC, GS, KZ, BNL and JM analyzed the data and modified the manuscript. All authors contributed to the article and approved the submitted version.

## Funding

This work was supported by grants from the National Natural Science Foundation of China (grant number 81902619 and 82173068) and Wuhan Shuguang Project (grant number 2022020801020447).

## Conflict of interest

The authors declare that the research was conducted in the absence of any commercial or financial relationships that could be construed as a potential conflict of interest.

## Publisher’s note

All claims expressed in this article are solely those of the authors and do not necessarily represent those of their affiliated organizations, or those of the publisher, the editors and the reviewers. Any product that may be evaluated in this article, or claim that may be made by its manufacturer, is not guaranteed or endorsed by the publisher.
