# Supplementary material for: A predictive signature based on enhancer RNA associates with immune infiltration and aids treatment decision in clear cell renal cell carcinoma
Source: Front Oncol. 2022 Oct 12;12:964838. doi: 10.3389/fonc.2022.964838 (PMC9597358; doi:10.3389/fonc.2022.964838)
Supplement: Supplementary file 1 [file Presentation_1.pptx]

## Slide 1
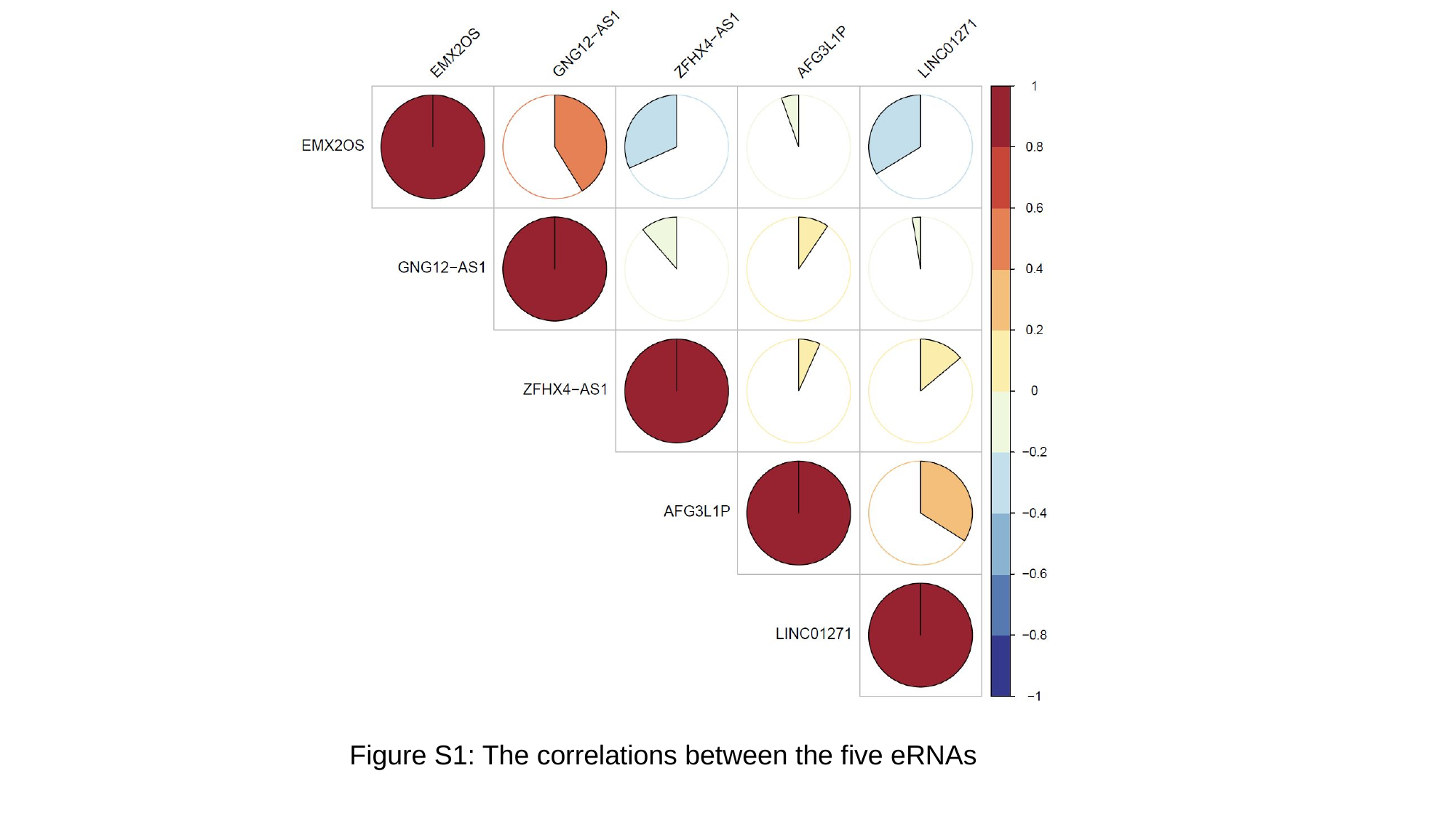

Figure S1: The correlations between the five eRNAs

## Slide 2
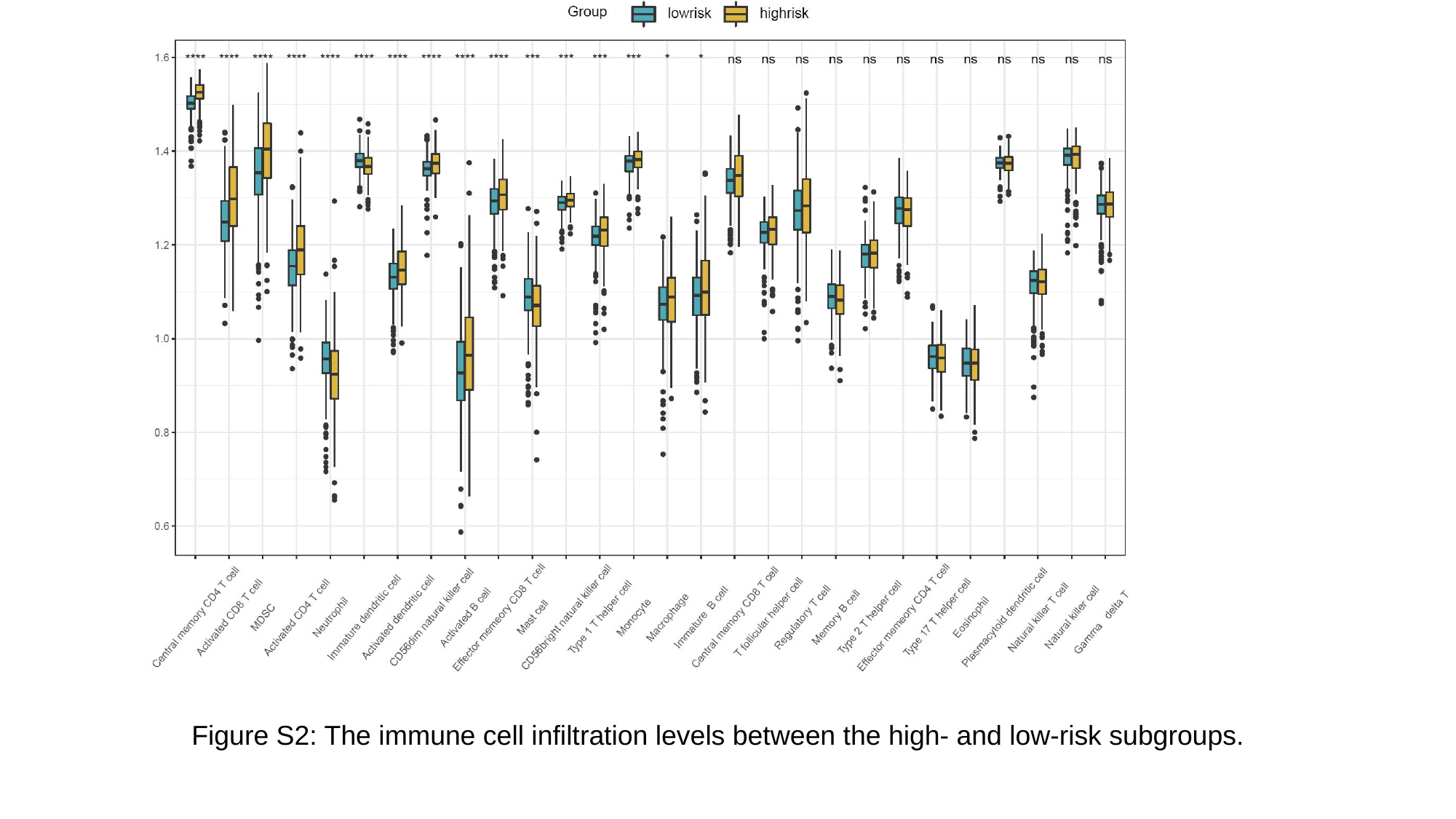

Figure S2: The immune cell infiltration levels between the high- and low-risk subgroups.

## Slide 3
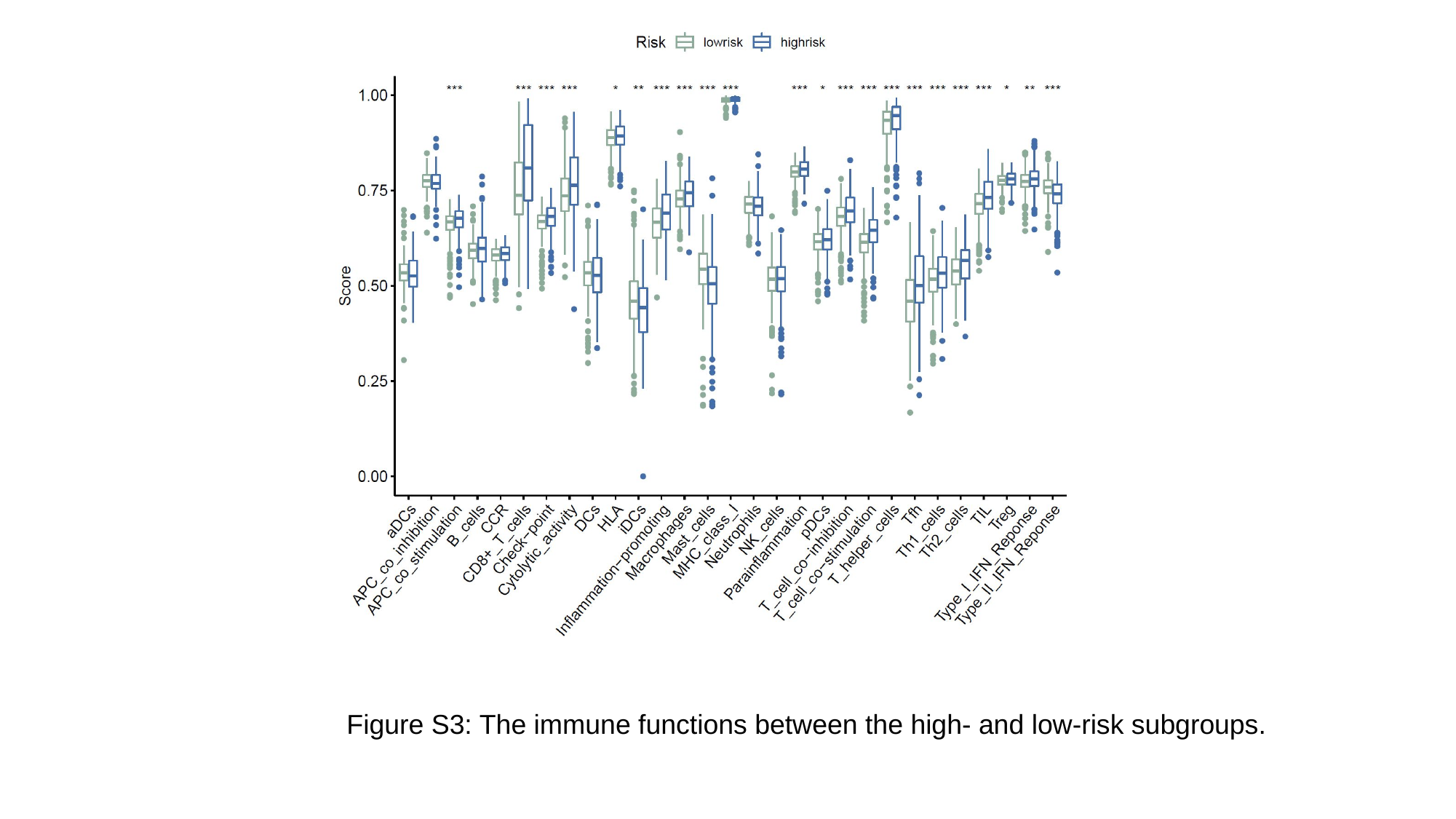

Figure S3: The immune functions between the high- and low-risk subgroups.
